# Influence of Habitat and Intrinsic Characteristics on Survival of Neonatal Pronghorn

**DOI:** 10.1371/journal.pone.0144026

**Published:** 2015-12-02

**Authors:** Christopher N. Jacques, Jonathan A. Jenks, Troy W. Grovenburg, Robert W. Klaver

**Affiliations:** 1 Department of Natural Resource Management, South Dakota State University, Brookings, South Dakota, United States of America; 2 U. S. Geological Survey, Iowa Cooperative Fish and Wildlife Research Unit, Iowa State University, Ames, Iowa, United States of America; University of Sydney, AUSTRALIA

## Abstract

Increased understanding of the influence of habitat (e.g., composition, patch size) and intrinsic (e.g., age, birth mass) factors on survival of neonatal pronghorn (*Antilocapra americana*) is a prerequisite to successful management programs, particularly as they relate to population dynamics and the role of population models in adaptive species management. Nevertheless, few studies have presented empirical data quantifying the influence of habitat variables on survival of neonatal pronghorn. During 2002–2005, we captured and radiocollared 116 neonates across two sites in western South Dakota. We documented 31 deaths during our study, of which coyote (*Canis latrans*) predation (*n* = 15) was the leading cause of mortality. We used known fate analysis in Program MARK to investigate the influence of intrinsic and habitat variables on neonatal survival. We generated a priori models that we grouped into habitat and intrinsic effects. The highest-ranking model indicated that neonate mortality was best explained by site, percent grassland, and open water habitat; 90-day survival (0.80; 90% CI = 0.71–0.88) declined 23% when grassland and water increased from 80.1 to 92.3% and 0.36 to 0.40%, respectively, across 50% natal home ranges. Further, our results indicated that grassland patch size and shrub density were important predictors of neonate survival; neonate survival declined 17% when shrub density declined from 5.0 to 2.5 patches per 100 ha. Excluding the site covariates, intrinsic factors (i.e., sex, age, birth mass, year, parturition date) were not important predictors of survival of neonatal pronghorns. Further, neonatal survival may depend on available land cover and interspersion of habitats. We have demonstrated that maintaining minimum and maximum thresholds for habitat factors (e.g., percentages of grassland and open water patches, density of shrub patches) throughout natal home ranges will in turn, ensure relatively high (>0.50) neonatal survival rates, especially as they relate to coyote predation. Thus, landscape level variables (particularly percentages of open water, grassland habitats, and shrub density) should be incorporated into the development or implementation of pronghorn management plans across sagebrush steppe communities of the western Dakotas, and potentially elsewhere within the geographic range of pronghorn.

## Introduction

Populations of large herbivores respond to changes in abundance or behavior of predators, disease prevalence, or anthropogenic activities [1]. Consequently, temporal variation in ecologic or anthropogenic factors should prominently influence population dynamics and demographics of these species [2–3]. Despite these potential sources of heterogeneity in survival rates, populations of large herbivores often are considered weakly affected by temporal variation [4]. Regardless of the source of variability, pronounced differences in inter-annual variation in survival exist along a continuum, with little year-to-year variation in adult female cohorts and strong year-to-year variability in juvenile age classes [1]. As such, the juvenile age class is considered the critical component of ungulate population dynamics [1]. Hence, survival and mortality rates are important parameters influencing many aspects of the management of mammal populations [5, 6].

Increased understanding of mortality of pronghorn (*Antilocapra americana*) is a prerequisite to successful management programs, particularly as it relates to population dynamics and the role of population models in adaptive species management [7]. Neonates (<1 month of age; [8]) are the life stage whereby pronghorn are most vulnerable to mortality [9]. Therefore, obtaining reliable survival estimates is critical for understanding how prehunting season survival rates affect annual recruitment and thus, pronghorn herd sustainability and associated harvest strategies [7, 10]. Consequently, site-specific studies investigating survival and cause-specific mortality of neonatal pronghorn have been conducted across the species’ geographic range [8, 11].

Previous research indicates that pronghorn mortality is attributable to a myriad of factors that vary spatially and temporally with respect to sex and age classes, and pronghorn density [11]. A primary source of mortality includes predation on neonates [8, 12–13], which has been associated with a wide range of intrinsic mechanisms. For instance, timing of birth may affect vulnerability of neonatal pronghorn to predation given their tendency to lie in seclusion during the first few weeks of life [14–15]. Pronghorn exhibit tightly synchronized birth dates, most notably across the northern portion of their range, which is thought to be a behavioral mechanism to reduce loss of young to predation [12–13]. However, some evidence suggests that neonates born during the peak of the birth season may be at greater risk of predation than individuals born at other times during the fawning season [16]. Also, maternal condition late in gestation may affect birthweight in ungulates [17], which may increase susceptibility of neonates to hypothermia and starvation, or increase vulnerability to predation [18–19]. Additionally, Byers and Moodie [20] documented intersexual differences in activity level in neonatal pronghorn, with females being more active than males early in life.

Spatial heterogeneity in security cover at neonatal pronghorn bedding sites has been one of the most investigated aspects of interrelationships between pronghorn and microhabitat quality since the 1970s [21]. Moreover, it is widely thought that macrohabitat selection by parturient females possibly determines microhabitat site characteristics at neonatal bedsites [22– 23], though research linking macrohabitat site selection to survival of pronghorn neonates is lacking. However, studies of other North American ungulate species have confirmed that macrohabitat features affect survival of neonatal woodland caribou (*Rangifer tarandus*; [24]), mule deer (*Odocoileus hemionus*; [25], moose (*Alces alces*; [26]), elk (*Cervus elaphus*; [27]), and white-tailed deer (*O*. *virginianus*; [28–29]), and such is likely the case for neonatal pronghorns.

Despite being poorly understood throughout sagebrush (*Artemisia* spp.) steppe communities of the western U.S., inter-annual and site-specific variability in birth weight, age, parturition date, and sex may be important factors affecting survival of neonatal pronghorn.

To our knowledge, only Jacques et al. [8] has quantified survival and cause-specific mortality of neonatal pronghorns across the rangelands of western South Dakota, whereby notable differences in survival rates were documented between study sites; despite similarities in predator suites, regional differences in neonatal survival was likely associated with variation in coyote relative abundance. Nevertheless, their analyses did not investigate potential effects of habitat and intrinsic variables on survival. Presumably, lower quality habitats across regions within the eastern-most extension of sagebrush-steppe communities may contribute to lower survival of neonatal pronghorn than in regions characterized by higher quality fawning habitat further west. Thus, our objective was to determine the site-specific influence of habitat and intrinsic characteristics on survival of pronghorn neonates across sagebrush-steppe communities of western South Dakota. Because of the association of habitat characteristics with fawn recruitment and subsequent sustainability of pronghorn populations, we hypothesized that habitat variables would have a greater effect on neonatal survival than intrinsic factors. Evaluation of this hypothesis will contribute to development of a benchmark for understanding potential effects of site-specific habitat factors on pronghorn survival across sagebrush-steppe communities throughout the western U.S.

## Materials and Methods

### Study Area

**Fig 1:**
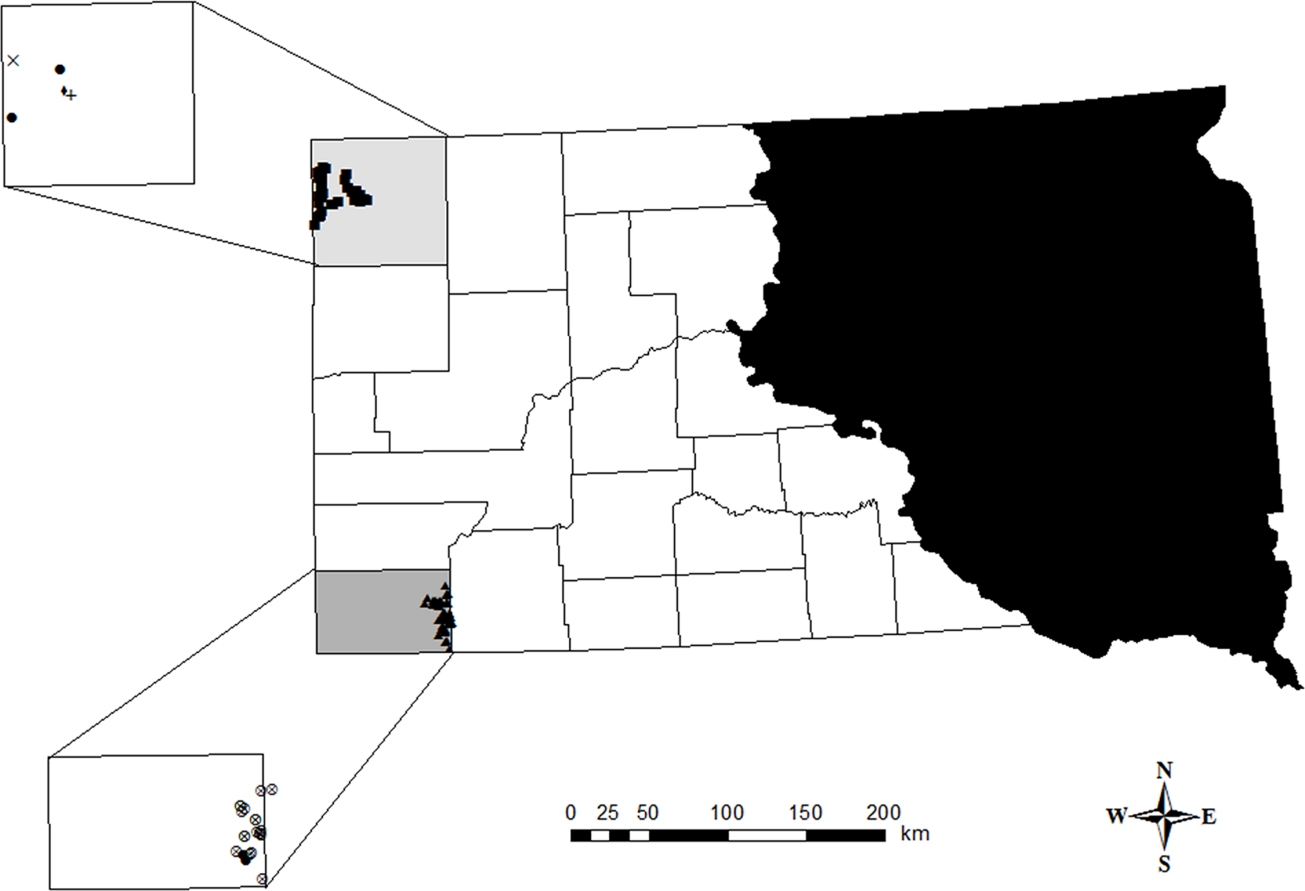
Neonatal pronghorn (*Antilocapra americana*) capture locations were located in Harding (gray shaded county in northwest region of state) and Fall River (light gray shaded county in southwest region of state) of western South Dakota, 2002–2005. Thin black lines delineated county boundaries and the black shaded region encompassing eastern South Dakota represented the area outside current pronghorn range. ■ denotes Harding County neonatal capture locations. ▲ denotes Fall River County neonatal capture locations. ♦ = neonatal death by cattle trampling, ⊗ = neonatal death by predation, + = neonatal death by abandonment, • = neonatal death by unknown causes, and × = neonatal death by vehicle collision.

Our study was conducted in a 6,940-km^2^ area of northwestern (e.g., Harding County) and a 5,071-km^2^ area of southwestern (e.g., Fall River County) South Dakota (Fig 1). Landscape in western South Dakota was characterized by flat to gently rolling topography and a mosaic of mixed-grass prairie interspersed with sagebrush [30–31]. Distribution of pronghorns in western South Dakota was within an eastward extension of sagebrush-steppe communities, including big sagebrush (*A*. *tridentata*) and silver sagebrush (*A*. *cana*) [32–33]. Regional coyote densities were unknown across western South Dakota, though moderate to intensive coyote management (aerial shooting and state trappers responding to landowner complaints) was conducted throughout Harding and Fall River counties monthly from 2002 through 2005 [8].

The majority (75%) of land area in Fall River County was characterized as grassland grazed by livestock, whereas the remaining acreage constituted shrub (13%), forest (6%), cultivated crops (3%), development (1.4%), wetlands (1%), and open water (0.5%) [34]). Land elevation ranged between 914 m and 1,478 m above mean sea level. Fall River County was located within the mixed grass prairie region of western South Dakota and dominant grasses on the landscape included western wheatgrass (*Agropyron smithii*), buffalograss (*Buchloe dactyloides*), green needlegrass (*Stipa viridula*), needle-and-thread (*S*. *comata*), side oats grama (*Bouteloua curtipendula*), blue grama (*B*. *gracilis*), and prairie junegrass (*Koeleria macrantha*). Dominant overstory woody vegetation consisted of limited stands of ponderosa pine (*Pinus ponderosa*) interspersed with small stands of quaking aspen (*Populus tremuloides*) and paper birch (*Betula papyrifera*; [35]). Silver sagebrush and big sagebrush were limited in distribution throughout Fall River County. Plant nomenclature followed Larson and Johnson [36] and Johnson and Larson [37].

Most of the land area in Harding County was treeless, semi-arid rolling plains. Land elevation ranged between 817 m and 1,224 m above mean sea level and 81% was characterized as grassland used as grazing land. Remaining acreage constituted shrub (6%), forest (1.4%), cultivated crops (6.4%), development (0.8%), wetlands (0.7%), and open water (0.3%; Homer et al. 2007). Dominant grasses on the landscape included western wheatgrass, prairie junegrass, buffalograss, green needlegrass, and blue grama. Silver sagebrush and big sagebrush were the dominant shrubs on the landscape [38].

### Pronghorn Capture and Monitoring

We captured neonatal pronghorns using an observation method described by Byers [12] and fitted them with expansion breakaway radio collars (Advanced Telemetry Systems, Isanti, Minnesota, USA) during late May and early June 2002 to 2005. Our capture efforts occurred on private land, and the owner of each parcel granted us permission to capture. The South Dakota Department of Game, Fish and Parks granted us permission to capture neonatal pronghorn on state lands. We did not conduct capture activities on tribal lands and endangered species were not captured. We recorded sex, weight, and new hoof growth measurements (mm) of captured neonates. We estimated age (days) of each neonate by examining hoof characteristics, noting neonate behavior, and recording umbilicus condition [39–40]. The Institutional Animal Care and Use Committee at South Dakota State University specifically approved this study (approval number 02-A0001), including all capture, animal handling, and sampling methods. Our animal handling methods followed guidelines for the care and use of animals approved by the American Society of Mammalogists [41].

We monitored survival status of radio-collared neonates 2–3 times per day (e.g., early morning, mid-day, late afternoon) until approximately 13 weeks post-capture through the end of each summer field season (31 August) using a vehicle mounted “null-peak” antenna system [42], hand-held directional antennas (Telonics Telemetry Electronic Consultants, Mesa, Arizona, USA), and fixed-wing Cessna aircraft (Cessna Aircraft Company, Wichita, Kansas, USA). When we detected a mortality signal, we immediately (<1 hr) located the collar, conducted field necropsies, and recorded evidence at mortality sites to determine the likely cause of death [43]. Given our daily monitoring intensity, mortality events occurred ≤12 hrs before investigating death sites. If collars were prematurely shed, we right-censored neonates from analyses at the time of collar loss [44]. We classified mortality events as unknown if cause of death could not be determined in the field, and subsequently transported animals to the Animal Disease Research Diagnostic Laboratory (ADRDL) at South Dakota State University for further examination [8, 43].

### Data Analyses

To evaluate potential effects of habitat characteristics on neonate survival, we created 1,225-m and 1,720-m circular analysis regions around initial capture locations [45–46]; associated circular analysis regions (4.71 km^2^ and 9.29 km^2^, respectively) comprised a land area that was the approximate size of the mean 50% summer home range of neonatal pronghorns across Harding and Fall River counties, respectively. We conducted our analyses using 50% natal home ranges due to the hiding tendency and sedentary behavior of neonatal pronghorn during the first 3 weeks of life [14]. Further, most mortality among neonatal pronghorn occurs within 3-weeks of birth [13, 47–49]; thus, predisposition of individuals to increased mortality is likely associated with habitat characteristics at smaller spatial scales (i.e., 50% natal home ranges). Moreover, circular analysis regions encompassed the entire area used by animals prior to most (76%) mortality, thus were likely reflective of true 50% natal ranges rather than core areas within those ranges. To determine habitat characteristics associated with each neonate, we overlaid circular analysis regions on the 2006 National Land Cover Data set (NLCD; [34]) and calculated habitat composition (% composition of each buffer) using Geospatial Modeling Environment (http://www.spatialecology.com/gme) in ArcGIS 10.3 (Esri, Inc., Redlands, California, USA). We re-classified land cover data into 9 categories; grassland, pasture-hay, cultivated crops, barren land, development, forested cover, shrubs, wetlands, and open water. For a detailed description of land cover categories, see the NLCD website (http://www.mrlc.gov/nlcd06_leg.php). We used FRAGSTATS Version 4.2 to calculate landscape and class-level metrics associated with each buffered area by county [50].

We selected the initial set of habitat factors (14 variables; Tables 1 and 2) and intrinsic factors (6 variables; Table 3) that we considered biologically meaningful to neonatal ecology. Further, these variables also have been identified as important factors influencing neonatal survival of other ungulate species by affecting distribution, density, and hunting efficiency of predators [29]. We broadly defined habitat variables as a) percent cover (percent of landscape comprised of habitat cover type), b) patch density (number of patches/100 ha of the cover type), c) shape index (i.e., average departure of patches from maximum compaction), and d) landscape shape index (i.e., standardized measure of the edge for all cover type patches; [50]). To minimize potential confounding effects of heterogeneity in size of circular analysis regions between study sites on survival estimates, we excluded mean shrub, open water, and grassland patch area variables (Table 1) from our analyses. We included capture year, age at capture, birth mass, sex, and parturition date as intrinsic covariates in survival models. Though relative coyote densities were unknown across our study sites, we included study site (e.g., proxy for relative coyote density) as an intrinsic covariate in survival models to test whether differing coyote densities resulted in differential mortality due to predation or whether regional variation in coyote densities influenced habitat selection by neonatal pronghorn. Prior to modeling, we screened all independent variables for collinearity using Pearson’s correlation coefficients (|r| > 0.5) and used quantile plots to evaluate assumptions of normality [51]. We used only 1 variable from a set of collinear variables for modeling, which resulted in 9 uncorrelated habitat variables and 6 intrinsic variables used to evaluate potential effects of habitat and intrinsic factors on neonate survival (Tables 1–3).

**Table 1:** Final variables we quantified within capture areas used for evaluating potential habitat effects on pronghorn neonatal survival in western South Dakota, USA, 2002–2005.

Variable name	Description
**Grassland cover**	Total grassland cover (%; grass)
**Cropland cover**	Total cropland cover (%)
**Open water**	Total open water (%; ow)
**Shrub cover**	Total shrub cover (%; shrub)
**Mean grassland patch area**	Average patch size (ha) for all grassland patches
**Mean open water patch area**	Average patch size (ha) for all open water patches
**Mean shrub patch area**	Average patch size (ha) for all shrub patches
**Grassland patch density**	Density (no./100 ha) of all grassland patches (grass_pd)
**Cropland patch area**	Average patch size (ha) for all cropland patches
**Shrub patch density**	Density (no./100 ha) of all shrub patches (shrub_pd)
**Grassland shape index**	Average departure of grassland patches from maximum compaction (i.e., square shape; grass_si)
**Grassland patch index**	Percentage of landscape comprised by the largest grassland patch (grass_lpi)
**Landscape shape index**	Standardized measure of amount of edge adjusted for size of buffered area
**Mean patch area**	Average patch size (ha) for all habitat patches

**Table 2:** A priori candidate models constructed to determine potential effects of habitat variables on survival of neonatal pronghorn in western South Dakota, USA, 2002–2005.

Model^a^	K^b^	Description
***S***_**grass**_	2	% grassland cover influences survival
***S***_**ow**_	2	% open water influences survival
***S***_**shrub**_	2	% shrub cover influences survival
***S***_**grass + ow**_	3	% grassland cover and water influences survival
***S***_**grass_pd**_	2	Density of grassland patches influence survival
***S***_**shrub_pd**_	2	Density of shrub patches influence survival
***S***_**grass_si**_	2	Shape of grassland patches influence survival
***S***_**grass_si + grass_lpi**_	3	Shape and size of grassland patches influence survival
***S***_**full Kaplan-Meier**_	109	Survival was best explained by the fully saturated Kaplan-Meier model [55]

^a^Variables included in model defined in Table 1.

^b^Number of parameters.

**Table 3:** A priori candidate models constructed to determine potential effects of intrinsic variables on survival of neonatal pronghorn in western South Dakota, USA, 2002–2005.

Model	K^a^	Description
***S***_**constant**_	1	% grassland cover influences survival
***S***_**site**_	2	% open water influences survival
***S***_**age**_	2	% shrub cover influences survival
***S***_**parturition date**_ ^**b**^	2	% grassland cover and water influences survival
***S***_**birth mass**_	2	Density of grassland patches influence survival
***S***_**year**_	2	Density of shrub patches influence survival
***S***_**age + sex**_	3	Shape of grassland patches influence survival
***S***_**full Kaplan-Meier**_	109	Survival was best explained by the fully saturated Kaplan-Meier model [55]

^a^Number of parameters.

^b^Neonates were grouped into two categories, including peak-born and non-peak born.

To estimate survival to the end of the field season each summer (31 Aug) and determine factors influencing neonatal survival between survival and mortality areas, we used known fate models with the logit link function in Program MARK [52], which accommodated staggered entry and exit times of marked neonates during our analysis interval [53–54]. Additionally, Program MARK facilitated incorporation of model covariates in survival analyses, flexibility in model parameterization, and model selection [55]. To avoid potential effects of dependence among pronghorn siblings in survival analyses [55], we minimized the capture of twins during animal capture events. Given the small number of twins (*n* = 6) in our sample, we did not evaluate potential effects of sibling dependence on survival. Further, we followed the recommendations of Grovenburg et al. [56] to minimize potential effects of variation in age estimates on neonatal survival. Prior to analyses, we posited biologically plausible models of how neonatal survival might be influenced by habitat and intrinsic factors. We used Akaike’s Information Criterion corrected for small sample size (AIC_c_) to select a suite of models that best described the data. Our model set consisted of 15 *a priori* models (Tables 2 and 3) that grouped logically into habitat and intrinsic effects. We used the best approximating intrinsic model as the underlying (constant) structure for all habitat models to account for maximum variation in the data [57]. We compared AIC_c_ values to select the most parsimonious model and considered models differing by ≤2 ΔAIC_c_ from the selected model as competitive alternatives [57]. We used Akaike weights (*w*_*i*_) as a measure of relative support for model fit and multi-model inference to generate unconditional parameter estimates [57]. Parameter estimates were unconditional in the sense that they were conditioned on a suite of models weighted by their respective AIC_c_ scores rather than on a single best model where probability of being the optimal model may be only marginally higher than lower-ranked models [57]. Additionally, we examined models differing by ≤2 ΔAIC_c_ from the highest-ranked model to determine if they differed by 1 parameter from the best model and whether they had essentially the same maximized log likelihood [57–58]. In this case, models containing uninformative parameters were noncompetitive because they contributed an additional parameter that had no explanatory ability [57–58]. We used model averaging to account for model selection uncertainty [57].

We evaluated the relative support for competitive model covariates by determining whether parameter estimates had associated confidence intervals (CIs) that included zero [43, 58–59]; parameters with CIs including zero were eliminated from model consideration. Because there is no goodness-of-fit test for classical known-fate data, we fit a fully saturated model and used the reduction of the global model to biologically plausible models to assess GOF [60]. We further evaluated model robustness by artificially inflating ĉ (e.g., model term describing overdispersion) from 1.0 (no dispersion) to 3.0 (extreme dispersion) to simulate a continuum of levels of dispersion reflected in Quasi-AIC_c_ (QAIC_c_; [43, 59, 61].

## Results

We captured and radio-collared 116 neonates (65 females, 51 males; 58 Harding County, 58 Fall River County), of which 90% (*n* = 104) were single neonate captures and the remaining individuals were twin captures. Peak parturition occurred on 25 May 2002, 25 May 2003, 19 May 2004, and 23 May 2005. Mean age at capture was 4.1 days (SE = 0.2, *n* = 116) and ranged from <1 to 14 days; 81% of neonates were ≤5 days of age at capture. Similarly, mean body weight at capture was 4.1 kg (SE = 0.1, *n* = 116), though it ranged from 2.8 to 6.2 kg. We documented 31 neonate deaths during our study (22 Fall River County, 9 Harding County); predation was the leading cause of mortality and accounted for 18 (58%; 15 attributed to coyote [*Canis latrans*] predation; 1 bobcat [*Lynx rufus*] predation, 1 badger [*Taxidea taxus*] predation, 1 likely coyote predation but unconfirmed) neonate mortalities (i.e., 16.4% of non-censored individuals); all predation events occurred in Fall River County (Fig 1) and were spatially distributed across grassland habitats. We attributed remaining deaths to capture-related factors (*n* = 6), cattle trampling (*n* = 1), vehicle collisions (*n* = 1), abandonment (*n* = 1), and unknown causes (*n* = 4; Fig 1). Because pronghorn exhibit close to the highest known level of maternal reproductive effort among ungulates [62], we assumed that natural abandonment was minimal. Further, we assumed the death of a late-born neonate (25 Jun) by a 1.5 yr-old (and presumably inexperienced) female during prolonged drought conditions was due to natural abandonment rather than capture-related activities. Necropsies conducted at the ADRDL revealed starvation as the likely cause of death for individuals that we attributed to unknown causes. We censored all capture-related deaths (*n* = 6) from survival analyses.

Our analysis indicated that confidence intervals for 5 of 6 intrinsic model parameters encompassed zero, suggesting that most intrinsic factors (sex, year, parturition date, birth mass, age) were not important predictors of survival of neonatal pronghorns. However, the site covariate was included in all 7 top-ranked models, indicating it was an important predictor of neonatal survival (Table 4). Thus, we used the site covariate as the constant structure for our habitat models. Our analysis of habitat covariates revealed model selection uncertainty among competing survival models; weight of evidence (*w*_*i*_) supporting the best model {S_site + grass + ow_} (site, percent grass, and open water) was 0.41 (Table 4). The weight of evidence supporting this model was 1.3 times greater than the {S_site + ow_} (site, percent open water) model (*w*_*i*_ = 0.32), 4.2 times greater than the {S_site + shrub_pd_} (site, shrub patch density) model (*w*_*i*_ = 0.10), and 7.8 times greater than the {S_site +grass_si_} (site, grassland shape index) model (*w*_*i*_ = 0.05; Table 4). All other models were considered non-competitive (*w*_*i*_ < 0.12) and thus, excluded from further consideration. For model {S_site + grass + ow_}, β and 90% confidence intervals for the intercept (10.86, SE = 2.39, 90% CI = 6.93–14.79), site (–1.47, SE = 0.55, 90% CI = –2.37 to –0.57), percent grassland (–0.04, SE = 0.03, 90% CI = –0.08 to –0.01), and percent open water (–1.30, SE = 0.41, 90% CI = –1.98 to –0.63) indicated that β ≠ 0, indicating these factors were important predictors of neonatal survival. For model {S_site + shrub_pd_}, β and 90% confidence intervals for the intercept (5.99, SE = 0.59, 90% CI = 5.01–6.97), site (–1.31, SE = 0.52, 90% CI = –2.16 to –0.45), and shrub patch density (0.21, SE = 0.12, 90% CI = 0.02–0.40) indicated that β ≠ 0, indicating that density of shrub patches also was an important predictor of neonatal survival. Similarly, for model {S_site + grass_si_}, β and 90% confidence intervals for the intercept (6.12, SE = 0.67, 90% CI = 4.80–7.44), site (–1.53, SE = 0.51, 90% CI = –2.53 to –0.53), and grassland shape (0.16, SE = 0.11, 90% CI = 0.02–0.33) indicated that β ≠ 0, indicating that grassland patch also was an important predictor of neonatal survival. The remaining habitat covariates in the top-ranked models (Table 4) encompassed zero, indicating these factors were not important predictors of neonatal survival. Model {S_site + grass + ow_} had the lowest AIC_c_ when ĉ = 1.0 (no dispersion), though models {S_site + ow_} and {S_site_} had the lowest QAIC_c_ when ĉ = 2.0 (moderate dispersion; QAIC_c_ = 172.27) and ĉ = 3.0 (extreme dispersion; QAIC_c_ = 116.80), respectively. Using model {S_site + grass + ow_}, the 90-day survival rate was 0.80 (90% CI = 0.71–0.88) when mean percentages of grassland and open water were 86.16 and 0.38, respectively. Similarly, model-averaged 90-day survival using model {S_site + shrub_pd_} was 0.78 (90% CI = 0.70–0.87) when mean percentage of shrub density was 3.62 shrub patches per 100 ha.

**Table 4:** Top-ranked survival models relative to fully saturated Kaplan-Meier model for neonatal pronghorn from birth to 31 August in western South Dakota, USA, 2002–2005 from intrinsic and habitat covariates when ĉ (model overdispersion term) was 1.0 (i.e., assumes data are not overdispersed).

Model	K^a^	AIC_c_ ^b^	∆ AIC_c_ ^c^	W_i_ ^d^	Deviance
***S***_**site + grass + ow**_	4	338.02	0.00	0.41	330.02
***S***_**site + ow**_	3	338.53	0.50	0.32	332.52
***S***_**site + shrub_pd**_	3	340.87	2.85	0.10	334.87
***S***_**site *+* grass_si**_	3	342.14	4.12	0.05	336.14
***S***_**site**_	2	342.39	4.37	0.05	338.39
***S***_**full Kaplan-Meier**_	109	486.18	148.16	0.00	265.36

^a^Number of parameters.

^b^Akaike’s Information Criterion corrected for small sample sizes [57].

^c^Difference in AIC_c_ relative to minimum AIC_c_.

^d^Akaike weight [57].

**Fig 2:**
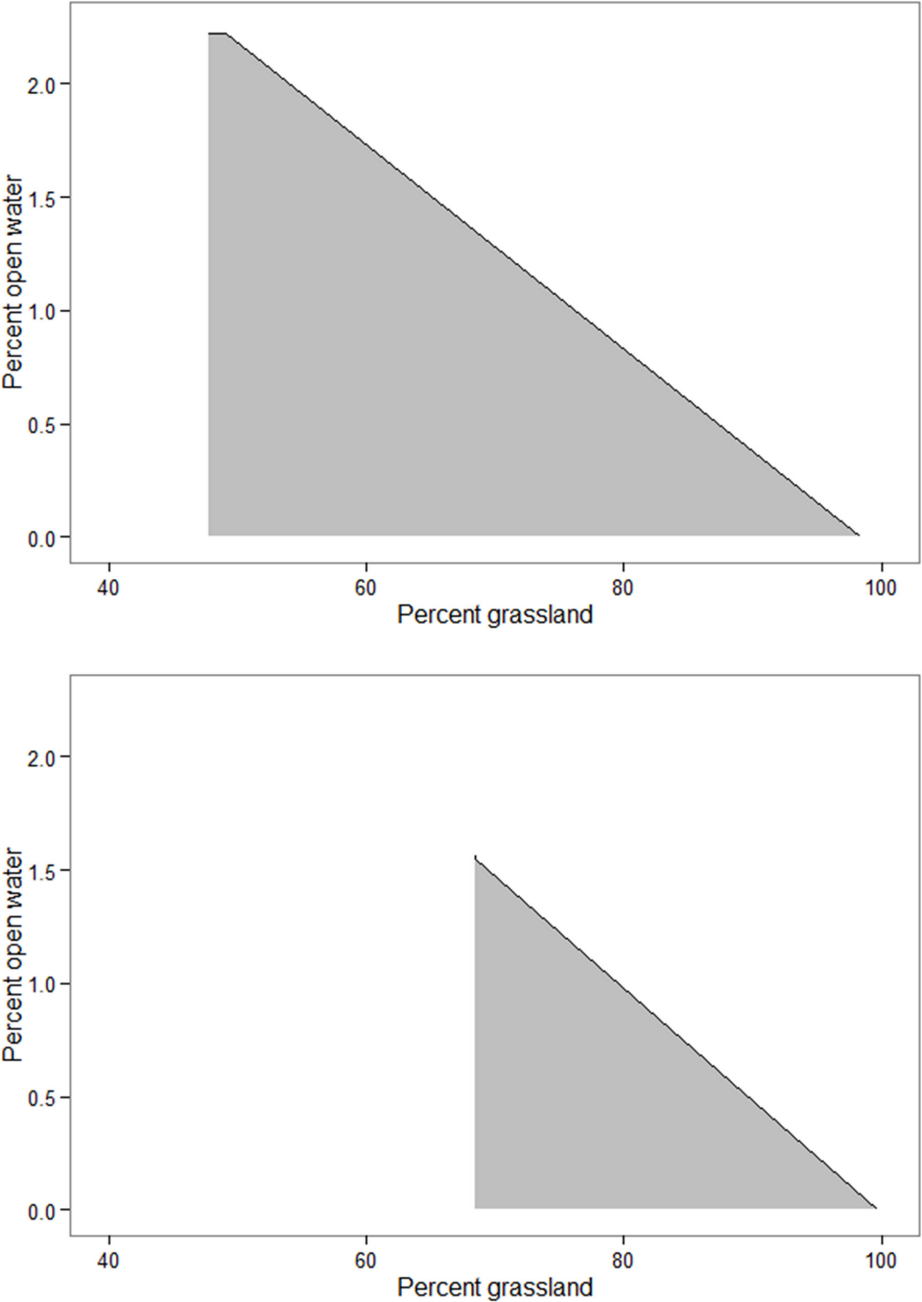
Relationship between percentages of open water and grassland on survival of neonatal pronghorn throughout western South Dakota, USA, 2002–2005. For any combination of grassland and open water percentages above the solid line, 90-day survival for Harding (top graph) or Fall River (bottom graph) counties declines below 0.50. The gray shaded area constitutes 90-day survival equal to or greater than 0.50.

Our results indicated that neonatal mortality increased 23% (0.87 to 0.71 survival) when grassland and water percentages throughout 50% natal home ranges increased from 80.1 to 92.3% and 0.36 to 0.40%. Similarly, mortality increased to >0.50 when home ranges within Harding and Fall River counties contained 48% and 68% grassland and 2.2% and 1.6% water, respectively (Fig 2). Moreover, mortality increased to >0.50 in Harding and Fall River counties when home ranges contained 98% and 100% grassland, respectively, and 0% water (Fig 2). When shrub density declined from 5.0 to 2.5 patches per 100 ha, mortality increased by 17% between Harding (0.15) and Fall River (0.27) counties; below 2.5 patches neonate mortality increased to ~0.50.

## Discussion

Survival of neonatal pronghorn was best described by two competing models containing site percentages of grassland and open water habitat, and shrub density throughout natal home ranges. Our findings confirm previous research whereby neonatal survival was related in part, to site-specific variation in coyote relative abundance [8]. Relative coyote densities were unknown within our study sites, though the number of animals removed over the study duration from Harding (1,457) and Fall River (1,128) counties was similar [8]. Nevertheless, predator control efforts were approximately 2 to 6 times greater in Harding than Fall River County, indicating relative coyote densities were higher in the southwestern region of the state. Consequently, predation was the primary cause of neonatal mortality, of which all coyote deaths occurred in Fall River County. Thus, it is possible the relative importance of habitat (e.g., percentages of grassland and open water, shrub density) covariates on neonatal survival were confounded by regional variation in coyote densities [8]. However, our results indicate that the site model (S_site_; proxy for coyote densities) was not substantial (Table 4) and that additive effects of grassland, open water habitat, and shrub density partially supports our hypothesis regarding the relative importance of habitat variables on survival of pronghorn neonates inhabiting grassland and sagebrush-steppe communities of western South Dakota.

Pronghorn populations typically demonstrate complex, age-structured population dynamics that are driven, in part, by climatic effects [31, 63]. For instance, previous research has described the importance of surface water and availability of grassland habitats on selection of bed sites [21, 64–65], though these studies did not evaluate whether habitat selection affected neonate survival. Limited mortality events precluded our ability to rigorously evaluate county-specific habitat factors on neonatal survival, though our analyses indicated that shrub density was an important predictor of neonate survival in Harding County. In contrast, percentages of grassland and open water habitats were important factors influencing neonatal survival in Fall River County. Despite spatial variation in habitat composition and coyote densities between study sites, our results indicated that neonatal pronghorn may exhibit functional responses in habitat use [66–70], whereby selection for habitat characteristics are altered depending on predator densities and availability of grassland, shrubs, and open water patches within natal ranges. Nevertheless, a paucity of published data needed to evaluate potential effects of habitat factors on survival of neonatal pronghorns makes our research unique.

Higher grassland cover and larger grassland patch size contributed to higher mortality of neonatal pronghorn, which is in contrast to previous studies of interrelations between habitat patch dynamics and survival of terrestrial vertebrates. Lower survival rates for nesting waterfowl have been attributed to smaller grassland patches and thus, increased predator search efficiency across the Prairie Pothole Region [71–73]. Further, Rohm et al. [29] noted higher white-tailed deer fawn survival in larger forest patches and a lower propensity of predators to search larger patches as frequently as smaller forest patches. Moreover, our results indicated that lower density of shrub habitats contributed to higher mortality of neonatal pronghorn, which is consistent with previous investigations that have described the importance of shrubs as concealment cover for neonatal pronghorn. For instance, Canon and Bryant [74] and Barrett [64] hypothesized that greater shrub density decreased the probability of detection of neonates by predators. Further, Jacques et al. [8] noted that regional variation in survival of neonatal pronghorn was associated, in part, with availability of vertical structure (e.g., shrub cover) at bed sites. Our results support previous investigations linking landscape complexity (e.g., associations between shrub patch density and grassland cover) with reduced visibility of pronghorns [75], which may have contributed to regional differences in survival of neonatal pronghorn across western South Dakota.

Our results suggested an association between grassland shape index and neonatal survival. Neonatal survival was influenced by grassland patch size and edge density, which is consistent with previous findings. For instance, Rohm et al. [29] noted increased survival of white-tailed deer fawns in areas with greater edge density, which they hypothesized as an indicator of higher quality habitat and suggested that more irregular forest patches characterizing survival areas may have affected the ability of predators to locate and capture neonates with nonlinear edges being more difficult to search than linear edges. Consequently, differences in habitat characteristics between summer home ranges of neonatal pronghorn likely involved complex interactions between habitat factors and density of predators. Despite searching larger grassland patches, fewer linear edges and reduced habitat complexity (e.g., lower shrub density) may have facilitated increased search efficiency by predators [8] and contributed to site-specific variation in neonatal survival during our study.

Despite some debate over the importance of abundant free standing water to artiodactyls [76], our results suggested the importance of open water as an important predictor of pronghorn neonate survival. Previous studies have noted seasonal shifts in home range use by ungulates in response to water [77]; parturient females occur closer to water during summer months than winter months and females and their offspring make extensive use of water during summer [78]. Hence, previous findings are consistent with the assumption that free water is important to meeting lactation demands of parturient females and their offspring [79]. Presumably, temporal home range shifts by predators in response to spatial distribution of prey species also occurs. Though uncertain, it is possible that increasing availability (%) of open water throughout neonatal summer home ranges may have contributed to higher relative densities of pronghorn during parturition and thus, higher relative densities of coyotes. Consequently, greater availability of water may have contributed to increased predation, and thus, regional variation in survival of neonatal pronghorn across western South Dakota.

Excluding the site covariate, variation in neonate survival was not explained by intrinsic covariates, which partially supports previous investigations of ungulate neonatal ecology. Though some evidence suggests that neonate survival increases with older and heavier males born farther from the peak of the birth season [80–82], other researchers have described potential benefits to neonates born prior to or at the peak of the birth season [83]. For instance, neonates born before peak birthing (and presumably before predators congregate on birthing grounds or before predators sharpen their search image) survive better than late-born neonates [83]. Similarly, neonates may benefit from being born during peak birthing due to the dilution effect [16] or increased vigilance and defense afforded by numerous parturient females [84–85]. Nevertheless, we were unable to detect any notable effects of intrinsic factors on survival of neonatal pronghorns. Though uncertain, the relative importance of intrinsic factors may have been a function of relatively few deaths observed during our study or the estimated age of neonates at capture. For instance, neonates ranged in age from <1 to 14 days at capture, with a majority of individuals (80%) captured within 5 days of birth. It is possible that a substantial amount of mortality had already occurred prior to capture, thereby minimizing potential effects of intrinsic covariates as meaningful predictors of neonatal survival. Alternatively, our inability to detect potential intrinsic effects on neonate survival may have been attributed to the spatial distribution of regional predation events across western South Dakota, all of which occurred in our Fall River County study site. Consequently, lack of spatial heterogeneity in predation events may have reduced spatial variability among intrinsic factors that often are accounted for in long-term studies [19]. Nevertheless, our results are consistent with Fairbanks [19], who noted little empirical support for potential effects of intrinsic factors (e.g., birth date, birth mass, sex, year) on survival of neonatal pronghorn. It is possible that with additional mortality events, intrinsic covariates may have been stronger predictors of neonatal survival given that the majority of deaths during our study were associated with coursing predators (e.g., coyotes). The overall neonate survival rate (0.80) we documented (using model S_site + grass + ow_) is within the range of survival rates (13–92%) previously documented for the species [8, 65, 86–88], and further implicates coyote predation as the primary cause of mortality on neonatal pronghorns throughout sagebrush-steppe communities of western South Dakota.

## Conclusions

Survival of neonatal ungulates drives population dynamics and influences harvest and habitat management strategies [7, 89]. As such, increased knowledge of the role of habitat characteristics on neonatal survival is critical. Our research is important because few studies have established ecological benchmarks for understanding the role of habitat factors, especially as they relate to coyote predation, on neonatal pronghorn across the geographic range of this species. We have demonstrated that maintaining minimum and maximum thresholds for habitat factors (e.g., percentages of grassland and open water patches, density of shrub patches) throughout natal home ranges will in turn, ensure relatively high (>0.50) survival rates. However, we encourage researchers to use caution when considering management strategies whereby availability and density of habitat factors important to neonate survival are maintained above or below set thresholds, which in turn may negatively affect summer survival rates. When neonatal survival is a function of site-specific habitat characteristics, focusing habitat manipulation efforts around creating or maintaining adequate site-specific thresholds for components of concealment cover (e.g., shrub patches) and water resources may contribute to greater reliability in survival and annual recruitment rates and thus, sustainable pronghorn harvest strategies. Our findings provide suggestions for optimizing habitat components and thus, summer survival for neonatal pronghorn across the eastern-most extension of sagebrush-steppe communities. However, our analytical approach can be applied to enhance survival of neonatal pronghorn across the geographic range of this species.

## Supporting Information

S1 File Raw data used to evaluate effects of habitat and intrinsic characteristics on survival of neonatal pronghorn throughout western South Dakota, USA, 2002–2005. (PDF)
