# Supplementary material for: Influence of Habitat and Intrinsic Characteristics on Survival of Neonatal Pronghorn
Source: PLoS One. 2015 Dec 2;10(12):e0144026. doi: 10.1371/journal.pone.0144026 (PMC4667974; doi:10.1371/journal.pone.0144026)
Supplement: S1 File — (PDF) [file pone.0144026.s001.pdf]

Fawn	County	Year	Capture Date	Last Day Alive	Censor Date
/*151.712 */	Harding	2002	31-May-02	25-Jun-02	
/*151.723 */	Harding	2002	24-May-02	2-Jan-05	2-Jan-05
/*151.734 */	Harding	2002	29-May-02	21-Apr-04	21-Apr-04
/*151.744 */	Harding	2002	27-May-02	17-Mar-04	17-Mar-04
/*151.755 */	Harding	2002	28-May-02	18-Nov-02	21-Nov-02
/*151.763 */	Harding	2002	23-May-02	12-May-04	12-May-04
/*151.773 */	Harding	2002	26-May-02	17-Oct-03	17-Oct-03
/*151.782 */	Harding	2002	30-May-02	16-Nov-02	20-Nov-02
/*151.794 */	Harding	2002	31-May-02	26-May-04	26-May-04
/*151.803 */	Harding	2002	27-May-02	27-Feb-04	27-Feb-04
/*151.812 */	Harding	2002	26-May-02	27-Aug-03	27-Aug-03
/*151.824 */	Harding	2002	29-May-02	23-Sep-03	23-Sep-03
/*151.835 */	Harding	2002	23-May-02	23-Jan-04	23-Jan-04
/*151.844 */	Harding	2002	5-Jun-02	21-Apr-04	21-Apr-04
/*151.853 */	Harding	2002	30-May-02	17-Mar-04	17-Mar-04
/*151.863 */	Harding	2002	28-May-02	25-Mar-03	25-Mar-03
/*151.874 */	Harding	2002	28-May-02	11-Jun-02	
/*151.883 */	Harding	2002	28-May-02	25-Jul-03	25-Jul-03
/*151.891 */	Harding	2002	29-May-02	27-Aug-03	27-Aug-03
/*151.902 */	Harding	2002	26-May-02	16-May-03	16-May-03
/*151.913 */	Harding	2002	28-May-02	10-Aug-02	
/*151.924 */	Harding	2002	29-May-02	25-Jul-03	25-Jul-03
/*151.934 */	Harding	2002	27-May-02	17-May-03	17-May-03
/*151.942 */	Harding	2002	28-May-02	27-Feb-04	27-Feb-04
/*151.954 */	Harding	2002	30-May-02	21-Apr-04	21-Apr-04
/*150.114 */	Harding	2004	23-May-04	31-Aug-05	31-Aug-05
/*150.856 */	Harding	2004	22-May-04	31-Aug-05	31-Aug-05
/*150.946 */	Harding	2004	26-May-04	31-Aug-05	31-Aug-05
/*151.377 */	Harding	2004	27-May-04	13-Jun-04	14-Jun-04
/*151.586 */	Harding	2004	21-May-04	31-Aug-05	31-Aug-05
/*151.666 */	Harding	2004	21-May-04	31-Aug-05	31-Aug-05
/*151.714 */	Harding	2004	23-May-04	31-Aug-05	31-Aug-05
/*151.723 */	Harding	2004	26-May-04	9-Jun-04	10-Oct-04
/*151.733 */	Harding	2004	22-May-04	31-Aug-05	31-Aug-05
/*151.743 */	Harding	2004	26-May-04	24-Oct-04	25-Oct-04
/*151.753 */	Harding	2004	22-May-04	20-Nov-04	20-Nov-04
/*151.764 */	Harding	2004	22-May-04	22-Aug-04	
/*151.774 */	Harding	2004	22-May-04	31-Aug-05	31-Aug-05
/*151.782 */	Harding	2004	23-May-04	31-Aug-05	31-Aug-05
/*151.804 */	Harding	2004	22-May-04	31-Aug-05	31-Aug-05
/*151.804b */	Harding	2004	25-May-04	31-Aug-05	31-Aug-05
/*151.813 */	Harding	2004	22-May-04	31-Aug-05	31-Aug-05
/*151.823 */	Harding	2004	23-May-04	1-Oct-04	02-Oct-04
/*151.834 */	Harding	2004	23-May-04	5-Nov-04	6-Nov-04
/*151.864 */	Harding	2004	22-May-04	31-Aug-05	31-Aug-05
/*151.874 */	Harding	2004	20-May-04	31-Aug-05	31-Aug-05

/*151.882 */	Harding	2004	23-May-04	28-Nov-04	29-Nov-04
/*151.894 */	Harding	2004	24-May-04	6-Jun-04	
/*151.904 */	Harding	2004	23-May-04	31-Aug-05	31-Aug-05
/*151.915 */	Harding	2004	22-May-04	31-Aug-05	31-Aug-05
/*151.934 */	Harding	2004	23-May-04	31-Aug-05	31-Aug-05
/*151.956 */	Harding	2004	21-May-04	31-Aug-05	31-Aug-05
/*151.712 */	Fall River	2003	29-May-03	14-Sep-03	15-Sep-03
/*151.723 */	Fall River	2003	26-May-03	30-May-05	30-May-05
/*151.733 */	Fall River	2003	30-May-03	14-Jun-03	
/*151.743 */	Fall River	2003	5-Jun-03	12-Jun-03	
/*151.755 */	Fall River	2003	31-May-03	2-Sep-04	07-Sep-04
/*151.763 */	Fall River	2003	1-Jun-03	31-Aug-05	31-Aug-05
/*151.775 */	Fall River	2003	31-May-03	24-Jun-04	24-Jun-04
/*151.783 */	Fall River	2003	30-May-03	29-Jun-04	29-Jun-04
/*151.793 */	Fall River	2003	30-May-03	31-Aug-05	31-Aug-05
/*151.803 */	Fall River	2003	2-Jun-03	5-Sep-04	05-Sep-04
/*151.813 */	Fall River	2003	27-May-03	28-Jun-03	
/*151.823 */	Fall River	2003	28-May-03	14-Jun-03	
/*151.834 */	Fall River	2003	31-May-03	26-May-04	26-May-04
/*151.844 */	Fall River	2003	26-May-03	28-Jul-05	28-Jul-05
/*151.854 */	Fall River	2003	27-May-03	15-Nov-04	15-Nov-04
/*151.864 */	Fall River	2003	31-May-03	19-Jun-03	
/*151.874 */	Fall River	2003	3-Jun-03	1-Oct-03	04-Oct-03
/*151.885 */	Fall River	2003	29-May-03	10-Feb-04	11-Feb-04
/*151.894 */	Fall River	2003	5-Jun-03	25-Jun-03	
/*151.904 */	Fall River	2003	29-May-03	12-Jun-03	
/*151.913 */	Fall River	2003	29-May-03	29-Jun-03	
/*151.923 */	Fall River	2003	29-May-03	14-Jul-03	
/*151.934 */	Fall River	2003	5-Jun-03	11-Oct-04	11-Oct-04
/*151.943 */	Fall River	2003	28-May-03	11-Jun-03	
/*151.954 */	Fall River	2003	30-May-03	31-Aug-05	31-Aug-05
/*151.096 */	Fall River	2005	26-May-05	31-Aug-05	31-Aug-05
/*151.096b */	Fall River	2005	1-Jun-05	31-Aug-05	31-Aug-05
/*151.377 */	Fall River	2005	25-May-05	31-Aug-05	31-Aug-05
/*151.515 */	Fall River	2005	1-Jun-05	31-Aug-05	31-Aug-05
/*151.712 */	Fall River	2005	3-Jun-05	31-Aug-05	31-Aug-05
/*151.723 */	Fall River	2005	26-May-05	31-Aug-05	31-Aug-05
/*151.743 */	Fall River	2005	26-May-05	31-Aug-05	31-Aug-05
/*151.755 */	Fall River	2005	22-May-05	31-Aug-05	31-Aug-05
/*151.764 */	Fall River	2005	29-May-05	31-Aug-05	31-Aug-05
/*151.775 */	Fall River	2005	26-May-05	14-Jun-05	
/*151.793 */	Fall River	2005	1-Jun-05	31-Aug-05	31-Aug-05
/*151.794 */	Fall River	2005	21-May-05	9-Jun-05	
/*151.803 */	Fall River	2005	22-May-05	16-Jun-05	
/*151.812 */	Fall River	2005	27-May-05	6-Jul-05	
/*151.823 */	Fall River	2005	27-May-05	31-Aug-05	31-Aug-05
/*151.835 */	Fall River	2005	29-May-05	17-Jun-05	

/*151.844 */	Fall River	2005	21-May-05	31-Aug-05	31-Aug-05
/*151.853 */	Fall River	2005	21-May-05	20-Jun-05	
/*151.874 */	Fall River	2005	24-May-05	3-Jul-05	
/*151.882 */	Fall River	2005	30-May-05	21-Jun-05	
/*151.885 */	Fall River	2005	26-May-05	31-Aug-05	31-Aug-05
/*151.894 */	Fall River	2005	24-May-05	31-Aug-05	31-Aug-05
/*151.902 */	Fall River	2005	27-May-05	31-Aug-05	31-Aug-05
/*151.913 */	Fall River	2005	27-May-05	9-Jul-05	
/*151.923 */	Fall River	2005	21-May-05	31-Aug-05	31-Aug-05
/*151.923b */	Fall River	2005	2-Jun-05	31-Aug-05	31-Aug-05
/*151.934 */	Fall River	2005	27-May-05	9-Jun-05	
/*151.942 */	Fall River	2005	27-May-05	31-Aug-05	31-Aug-05
/*151.942b */	Fall River	2005	1-Jun-05	31-Aug-05	31-Aug-05

Fate	Mortality Date	Age	Sex	Birth Mass	Part. Date	PLAND_GRASS	PD_GRASS
cattle tramping	06-Jun-02	3	0	2.80	1	47.757	3.851
		2	0	3.20	1	72.757	1.284
Lost Signal		6	0	4.10	1	81.748	0.856
		7	1	4.80	0	91.972	0.428
Dropped collar		3	1	3.70	1	91.509	0.642
Dropped collar		4	1	4.30	0	78.073	1.282
Lost Signal		2	0	2.90	1	65.774	2.782
		2	1	3.50	1	74.634	1.712
End of field season		7	0	4.30	1	69.309	1.710
Dropped collar		3	1	4.40	1	90.743	0.642
Dropped collar		4	1	4.40	1	74.264	1.497
Dropped collar		4	1	3.40	1	73.878	1.070
Dropped collar		3	1	3.90	0	67.238	1.712
Lost Signal		4	0	5.00	1	67.084	1.711
End of field season		2	0	3.10	1	59.245	2.140
Lost Signal		4	1	3.00	1	97.997	0.428
roadkill	12-Jun-02	3	0	3.00	1	96.338	0.214
Dropped collar		4	1	3.80	1	98.040	0.641
Dropped collar		13	0	4.90	0	57.319	2.996
Dropped collar		4	1	4.10	1	81.605	1.283
unknown	13-Aug-02	2	1	3.30	1	57.558	2.996
Dropped collar		3	1	3.90	1	70.044	1.495
Dropped collar		4	0	3.50	1	84.242	1.070
End of field season		2	0	2.80	1	86.418	1.069
Dropped collar		4	0	3.50	1	94.069	0.642
End of field season		5	0	5.60	1	94.742	0.428
End of field season		2	1	4.10	1	67.347	1.284
End of field season		3	1	3.60	0	91.742	0.428
Dropped collar		3	1	3.50	0	80.563	1.498
End of field season		5	1	4.50	1	84.527	0.428
End of field season		6	1	4.70	0	78.032	1.070
End of field season		6	0	4.20	1	73.613	1.284
Dropped collar		14	1	6.20	0	93.178	0.214
End of field season		4	1	5.10	1	90.853	0.428
		3	1	3.40	0	96.650	0.856
Dropped collar		3	1	3.30	1	67.617	1.925
unknown	23-Aug-04	4	0	3.60	1	89.024	0.428
End of field season		4	0	4.80	1	73.729	1.712
End of field season		3	0	3.60	1	79.134	0.642
End of field season		4	0	5.20	1	90.984	0.642
End of field season		7	1	5.40	1	88.771	1.284
unknown		4	1	4.80	1	73.719	1.285
End of field season		4	0	4.10	1	64.364	1.924
Dropped collar		2	0	3.00	1	75.327	0.428
End of field season		2	0	3.30	1	86.689	0.642
End of field season		5	1	4.50	0	83.946	0.642

Dropped collar		3	1	3.60	1	79.888	0.857
unknown	07-Jun-04	5	0	5.10	1	94.573	0.642
End of field season		3	1	3.60	1	83.937	0.856
		4	1	4.00	1	94.206	0.428
End of field season		7	0	5.00	1	70.243	1.712
End of field season		6	0	4.70	0	89.188	0.428
Dropped collar		3	1	3.60	1	77.921	0.430
Dropped collar		3	0	4.50	1	86.742	0.215
unknown	15-Jun-03	4	0	4.50	1	98.122	0.108
abdonment	13-Jun-03	5	1	4.50	1	92.484	0.215
		3	1	4.30	1	99.758	0.108
End of field season		8	0	5.80	1	94.075	0.215
Dropped collar		7	1	5.30	1	93.455	0.108
Dropped collar		3	1	3.10	1	88.525	0.108
End of field season		4	0	4.30	1	99.100	0.108
		7	0	5.00	1	94.693	0.215
predation	29-Jun-03	3	1	3.20	1	99.216	0.108
predation	15-Jun-03	4	1	4.30	1	94.442	0.108
Dropped collar		1	1	3.60	0	98.896	0.108
End of field season		4	0	4.30	1	95.111	0.215
		2	0	3.80	1	96.978	0.108
predation	20-Jun-03	4	0	3.50	1	92.661	0.108
		2	0	5.00	1	89.252	0.108
		10	0	5.70	0	96.649	0.215
predation	26-Jun-03	7	1	5.30	0	99.642	0.108
predation	13-Jun-03	4	0	3.50	1	99.564	0.108
predation	30-Jun-03	7	1	5.00	1	95.006	0.215
predation	15-Jul-03	4	1	4.10	1	92.085	0.323
		7	0	5.60	0	92.100	0.323
predation	12-Jun-03	5	0	4.40	1	98.828	0.108
End of field season		3	0	3.00	1	89.056	0.108
End of field season		3	0	3.30	1	68.458	1.076
End of field season		3	1	3.50	0	95.189	0.215
End of field season		6	0	4.20	0	96.466	0.108
End of field season		3	1	3.60	0	95.158	0.215
End of field season		5	0	4.50	0	90.822	0.215
End of field season		3	1	4.00	1	77.718	0.645
End of field season		3	1	4.30	1	79.483	0.861
End of field season		8	0	4.70	0	98.053	0.108
End of field season		2	1	4.30	0	81.487	0.323
predation	15-Jun-05	3	1	4.50	1	76.007	0.753
End of field season		5	0	4.20	1	94.685	0.430
predation	10-Jun-05	3	0	3.80	0	88.964	0.215
predation	17-Jun-05	4	1	4.30	0	93.773	0.215
predation	07-Jul-05	5	0	4.50	1	95.317	0.216
End of field season		1	0	3.60	1	84.468	0.430
predation	18-Jun-05	1	0	3.70	0	93.824	0.108

End of field season		4	0	4.20	0	95.045	0.108
predation	21-Jun-05	4	0	4.50	0	96.221	0.108
predation	05-Jul-05	3	0	3.80	1	89.988	0.215
predation	22-Jun-05	3	1	4.40	0	86.421	0.216
End of field season		7	1	4.80	0	82.076	0.215
End of field season		3	0	3.90	1	89.943	0.215
End of field season		5	0	4.70	1	96.718	0.108
predation	10-Jul-05	3	0	3.50	1	93.195	0.108
End of field season		4	0	4.60	0	95.199	0.108
End of field season		4	0	5.00	0	95.579	0.108
predation	10-Jun-05	3	0	4.20	1	93.320	0.108
End of field season		3	1	4.20	1	94.061	0.108
End of field season		3	0	3.80	0	94.829	0.108

LPI_GRASS	LSI_GRASS	PLAND_SS	PD_SS	LPI_SS	LSI_SS	PLAND_CROP	PLAND_OW	PD_OW
38.918	9.960	47.545	2.996	42.981	10.040	0.116	1.695	0.428
71.583	6.276	9.299	6.204	1.406	10.614	12.592	0.385	0.214
56.431	4.702	8.741	6.846	1.925	9.605	6.277	0.000	0.000
91.933	4.108	6.758	7.487	0.828	10.184	0.481	0.327	0.214
91.336	4.254	6.835	7.059	0.828	10.184	0.674	0.404	0.214
51.798	5.133	8.040	6.625	1.770	10.512	10.367	0.000	0.000
55.181	9.496	11.826	7.062	2.523	11.860	15.851	0.751	0.642
44.992	4.928	5.586	5.778	0.982	9.600	16.660	0.000	0.000
51.953	4.851	5.061	4.276	1.155	6.455	22.147	0.385	0.214
90.647	4.065	4.523	5.774	0.616	9.323	0.000	0.000	0.000
45.756	7.488	12.262	5.561	2.695	11.039	6.603	0.501	0.428
59.045	6.137	10.210	8.348	2.023	10.936	13.331	0.000	0.000
56.760	6.874	9.341	9.416	1.117	11.756	21.090	0.000	0.000
56.997	6.916	9.432	8.983	1.117	11.867	21.136	0.000	0.000
46.186	3.658	0.116	0.214	0.116	1.600	37.577	0.193	0.214
97.727	1.539	0.405	0.642	0.173	2.300	1.117	0.289	0.214
96.338	1.909	0.501	0.857	0.154	2.727	0.251	0.000	0.000
97.867	1.587	0.461	1.068	0.154	2.700	0.000	0.000	0.000
19.453	5.218	1.811	1.926	0.462	4.450	36.210	1.329	0.214
79.989	4.366	1.155	1.710	0.250	5.188	0.000	0.000	0.000
20.220	5.327	1.849	1.926	0.462	4.550	35.952	1.329	0.214
66.045	3.306	0.308	0.214	0.308	1.500	27.706	0.000	0.000
68.831	5.218	4.758	6.207	0.674	8.938	5.433	2.215	0.428
75.510	5.126	9.292	8.337	1.193	11.182	1.327	0.327	0.428
84.383	2.186	1.175	1.712	0.327	3.875	1.656	0.289	0.642
74.249	2.128	0.000	0.000	0.000	0.000	0.000	1.406	0.642
59.068	6.899	9.338	8.985	1.117	11.822	21.101	0.000	0.000
91.646	3.748	7.238	3.636	2.714	9.231	0.077	0.000	0.000
79.387	4.823	2.543	2.569	0.617	7.174	0.000	0.000	0.000
80.350	5.865	8.353	4.277	3.041	8.452	0.000	0.558	0.428
77.744	6.227	16.673	4.492	10.146	8.390	0.000	0.000	0.000
73.209	9.944	24.076	7.704	13.540	15.310	0.000	0.597	0.428
93.178	3.464	6.303	3.630	2.710	8.595	0.000	0.000	0.000
90.776	3.935	6.162	4.707	1.232	9.250	0.000	0.000	0.000
96.438	1.810	0.000	0.000	0.000	0.000	0.000	0.385	0.214
31.710	8.899	10.551	6.632	2.522	11.340	14.536	0.712	0.428
88.927	4.382	8.107	3.851	2.715	9.905	2.041	0.000	0.000
53.929	5.040	7.300	7.276	0.655	10.154	15.890	0.000	0.000
52.897	4.907	8.508	7.272	1.309	9.651	9.124	0.000	0.000
90.888	3.913	6.145	5.137	1.233	9.250	0.000	0.000	0.000
88.193	4.338	5.663	7.062	0.462	10.800	0.000	0.751	0.428
70.501	8.073	13.353	6.423	2.929	12.302	8.112	0.713	0.428
63.440	4.353	0.847	0.641	0.462	2.857	34.789	0.000	0.000
66.545	6.325	10.035	5.564	1.772	11.935	10.651	0.270	0.214
81.352	4.993	7.763	3.639	3.159	7.805	0.000	0.559	0.428
83.792	5.451	10.452	5.775	2.175	11.489	1.713	0.000	0.000

75.145	6.372	15.600	5.999	7.250	11.719	1.928	0.444	0.214
59.989	2.326	1.097	0.642	0.558	3.750	0.000	0.712	0.642
78.621	4.895	13.502	4.494	8.070	8.830	0.000	0.443	0.214
94.187	3.171	5.794	5.775	0.635	8.714	0.000	0.000	0.000
68.702	10.934	27.196	6.848	15.928	15.355	0.000	0.597	0.428
61.543	5.183	7.445	7.695	0.962	12.450	0.000	0.000	0.000
77.795	6.661	1.762	1.828	0.571	7.963	13.019	0.929	0.860
86.723	6.711	10.212	4.190	2.437	15.092	2.369	0.561	0.752
98.122	2.198	1.549	1.506	0.261	7.192	0.000	0.329	0.323
67.245	3.475	2.915	3.336	0.494	9.229	0.000	0.136	0.215
99.758	1.266	0.097	0.215	0.048	1.857	0.000	0.145	0.215
69.891	2.606	8.787	5.484	1.684	15.869	14.178	0.000	0.000
93.455	3.518	4.967	3.012	1.355	11.130	1.510	0.068	0.108
88.525	6.432	10.661	3.335	2.392	15.015	0.281	0.416	0.538
99.100	1.636	0.542	0.861	0.097	4.867	0.000	0.358	0.430
49.656	2.202	0.378	0.538	0.126	3.846	0.000	0.136	0.215
99.216	1.567	0.688	1.291	0.097	5.353	0.000	0.097	0.108
94.442	3.101	3.021	3.013	0.523	9.472	2.372	0.165	0.215
98.896	1.744	1.046	2.045	0.145	6.381	0.000	0.058	0.108
58.771	2.025	0.058	0.108	0.058	1.600	0.000	0.165	0.215
96.978	2.304	2.160	1.937	0.349	8.000	0.000	0.048	0.108
92.661	4.872	7.049	4.519	0.794	14.167	0.000	0.291	0.323
89.252	6.005	10.371	4.627	1.879	14.258	0.145	0.184	0.323
96.639	2.885	3.099	3.121	0.349	9.722	0.000	0.194	0.215
99.642	1.291	0.126	0.430	0.048	2.250	0.000	0.232	0.215
99.564	1.370	0.349	0.861	0.078	4.083	0.000	0.087	0.108
86.587	2.146	0.339	0.538	0.097	3.750	0.000	0.077	0.108
92.017	4.441	5.057	3.014	1.531	11.130	1.792	0.736	0.431
76.726	3.056	2.411	1.936	0.813	7.250	1.055	0.252	0.215
98.828	1.749	1.036	1.292	0.194	5.762	0.000	0.136	0.215
89.056	6.255	10.440	3.231	2.375	14.879	0.019	0.368	0.539
64.827	10.456	15.379	4.412	2.770	12.588	11.747	0.494	0.323
64.369	2.055	0.252	0.430	0.087	3.182	0.000	0.048	0.108
96.466	2.870	3.050	2.905	0.378	9.528	0.000	0.484	0.538
63.674	2.045	0.252	0.430	0.087	3.182	0.000	0.048	0.108
90.793	4.397	6.264	4.518	0.959	11.902	2.372	0.426	0.538
77.456	6.750	1.733	1.936	0.532	7.852	12.826	1.084	0.538
79.193	6.363	2.334	1.829	0.649	7.781	11.203	0.736	0.538
98.053	2.059	1.162	1.184	0.262	6.409	0.000	0.785	0.753
81.419	5.511	2.062	1.291	0.697	7.667	12.277	0.484	0.323
75.504	7.455	2.227	1.937	0.610	8.677	14.562	0.823	0.646
94.608	3.379	5.111	3.119	0.852	10.500	0.000	0.203	0.215
88.800	5.797	8.672	5.598	1.027	14.350	1.793	0.455	0.538
59.936	2.695	1.743	2.045	0.494	6.778	0.000	0.000	0.000
95.297	2.673	2.153	1.724	1.125	5.500	0.301	0.262	0.216
84.206	6.679	5.142	3.551	0.959	11.064	3.922	0.329	0.215
93.824	3.320	4.230	2.581	1.307	9.357	0.000	1.558	0.860

95.045	3.618	4.509	3.448	0.669	10.818	0.000	0.446	0.431
96.221	2.975	3.111	2.692	0.669	9.444	0.000	0.669	0.646
89.969	5.782	9.431	2.905	2.440	14.191	0.000	0.465	0.538
86.246	6.370	9.263	4.957	3.172	14.097	2.958	0.514	0.431
82.066	5.973	2.343	1.614	0.697	8.188	11.698	0.591	0.430
89.924	5.808	9.476	2.904	2.439	14.206	0.000	0.465	0.538
96.718	2.470	1.220	1.506	0.281	5.957	0.048	0.261	0.215
93.195	3.462	4.666	2.366	1.743	9.159	0.000	1.539	0.753
95.199	3.578	4.355	3.341	0.669	10.767	0.000	0.446	0.431
95.579	3.417	4.023	3.232	0.669	10.610	0.000	0.398	0.323
93.320	3.594	4.347	2.797	1.133	9.721	0.000	1.520	0.753
94.061	4.323	5.717	5.383	0.455	12.694	0.000	0.223	0.323
94.829	3.591	3.254	2.690	1.075	8.919	0.000	0.223	0.215

LPI_OW	PLAND_DEV
1.521	0.000
0.385	0.000
0.000	3.042
0.327	0.366
0.404	0.366
0.000	3.078
0.424	2.311
0.000	2.947
0.385	3.060
0.000	0.000
0.289	2.868
0.000	2.581
0.000	2.331
0.000	2.348
0.193	2.870
0.289	0.000
0.000	0.000
0.000	0.923
1.329	2.812
0.000	0.000
1.329	2.792
0.000	1.942
1.734	2.658
0.173	2.616
0.173	2.465
0.809	2.793
0.000	2.214
0.000	0.000
0.000	0.000
0.366	3.426
0.000	3.677
0.327	0.000
0.000	0.000
0.000	0.000
0.385	0.000
0.424	2.888
0.000	0.000
0.000	3.082
0.000	3.080
0.000	0.000
0.616	0.000
0.424	0.347
0.000	0.000
0.270	2.658
0.366	3.236
0.000	0.000

0.444	2.121
0.500	3.176
0.443	2.003
0.000	0.000
0.327	0.000
0.000	3.232
0.300	0.000
0.126	0.000
0.145	0.000
0.097	4.387
0.097	0.000
0.000	2.303
0.068	0.000
0.126	0.000
0.155	0.000
0.087	4.601
0.097	0.000
0.107	0.000
0.058	0.000
0.087	4.666
0.048	0.756
0.126	0.000
0.078	0.000
0.116	0.000
0.136	0.000
0.087	0.000
0.077	4.578
0.242	0.000
0.165	3.602
0.087	0.000
0.126	0.000
0.358	2.847
0.048	4.511
0.261	0.000
0.048	4.542
0.116	0.000
0.300	0.000
0.300	0.000
0.291	0.000
0.291	0.000
0.291	0.000
0.145	0.000
0.126	0.000
0.000	4.484
0.213	0.000
0.300	0.000
0.697	0.000

0.262	0.000
0.262	0.000
0.126	0.000
0.184	0.795
0.291	0.000
0.126	0.000
0.213	0.000
0.697	0.000
0.262	0.000
0.262	0.000
0.678	0.000
0.107	0.000
0.174	0.000
